# Applications of Chitosan, an Eco‐Friendly Biopolymer in Agricultural Systems, Herbal Products, and Functional Foods: A Review

**DOI:** 10.1002/fsn3.71367

**Published:** 2026-01-04

**Authors:** Saeedeh Karimlar, Abdollah Ghasemi Pirbalouti, Zahra Teymuori, Maryam Moslehishad, Zohreh Hamidi‐Esfahani

**Affiliations:** ^1^ Department of Food Science and Technology, Faculty of Agriculture Tarbiat Modares University Tehran Iran; ^2^ Translational Ophthalmology Research Center Tehran University of Medical Sciences Tehran Iran; ^3^ Department of Food Science and Technology TeMS.C., Islamic Azad University Tehran Iran; ^4^ Department of Food Science and Technology, College of Agriculture Isfahan University of Technology Isfahan Iran; ^5^ Department of Food Science and Technology ShQ. C., Islamic Azad University Shahr‐e Qods Iran

**Keywords:** elicitor, food industry, medicinal and aromatic plants, organic foods, secondary metabolites

## Abstract

Chitin, the natural biopolymer of the world next to cellulose, is a modified biodegradable polysaccharide. Chitosan, as the major derivative of chitin, is the product of N‐deacetylated chitin. Chitosan is an important biopolymer in nature and the only positively charged (cationic) polysaccharide. Chitosan has many utilizations in sustainable agriculture and food systems, in particular, improving plant resistance to environmental stresses like water deficit, salt, high temperature, cold, heavy metal, etc., as well as biotic stresses such as pest and plant pathogens. In addition, this natural biopolymer is used in different industries such as paper, food (processing, packaging, and preservation), pharmaceuticals, biodiesel, and other uses like wastewater treatment and environmental protection. Chitosan gained significant interest for its safety, antifungal, antibacterial, biodegradability, biocompatibility, and antioxidant activities due to its rich amino and hydroxyl groups. The commercial value of chitosan is due to the valuable properties of its soluble derivatives, which are suitable in food processing, cosmetics, nano and biotechnology, environmental, and textile production. In this review, we will consider the effectiveness of chitosan in the performance of agriculture, herbal products, nutraceuticals, and food systems, like improving biologically active compounds in herbal plants as elicitor; the characteristics of chitosan and chitosan‐based biopolymers have been mentioned.

## Graphical abstract

Chitosan is highlighted for its industrial applications include food processing and preservation, pharmaceuticals, agriculture systems, and environmental protection.

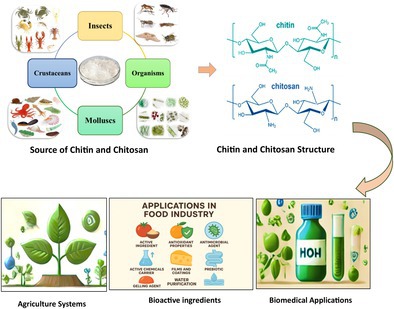

## 1 Introduction

Biopolymers as important macromolecules have been biosynthesized from living organisms such as plants, fungi, microbes, and animals, especially marine invertebrates and crustaceans. The main sources of chitin as a natural biopolymer and macromolecules can include insect biomass, algae, mushroom bodies, and microbial biomass (Ghasemi Pirbalouti et al. 2017). After cellulose, the second most abundant biopolymer in nature is the chitin that is extracted from crabs, shrimp, lobsters, and oysters (Hajji et al. 2022). Indeed, the shells and other unpalatable parts of these crustaceans, accounting for approximately half of their body mass, are often discarded waste (Kumar et al. 2019) as an important source of chitin. For example, chitin and its derivatives were found in the cell walls of four types of fungi including *Basidiomycetes*, *Ascomycetes*, *Zygomycetes*, and *Deuteromycetes* (Crognale et al. 2022). In addition, this biopolymer can also be found in plants. For instance, the content of chitin in the fruits of luffa or Egyptian cucumber or Vietnamese luffa ( *Luffa aegyptiaca* Mill.) belonging to the family Cucurbitaceae is less than in crustaceans like crab (Jiang et al. 2024).

Chitosan, a prominent derivative of chitin, was identified approximately 40 years after the discovery of its parent compound. This biopolymer is a polysaccharide consisting of N‐acetyl‐d‐glucosamine and d‐glucosamine units linked by β‐(1–4) glycosidic bonds (Wang et al. 2024). The functional groups present in chitosan, particularly amino (NH2) and hydroxyl (OH) groups, play a critical role in its diverse applications and appeal to researchers. Chitosan is widely recognized as a biodegradable, non‐toxic, non‐antigenic, and biocompatible natural polymer. This versatile substance belongs to a family of molecules characterized by variations in composition, molecular size, and monomer distribution. Additionally, chitosan exhibits notable chemical properties, including resistance to acids and thermal degradation. Chitin and chitosan have numerous applications and advantages. Chitosan is generally utilized in various industries, including food, pharmaceutical, cosmetic, and polymer industries due to its physical and chemical properties. In addition, chitosan is utilized in the clothing and paper industries (Aranaz et al. 2014; Younes and Rinaudo 2015). In the food industry, chitosan is valued for its antibacterial and antifungal properties, making it an effective preservative. It influences various functional properties, including protein aggregation, emulsification capacity, film‐forming ability, clarifying efficiency, and fatty acid absorption. These characteristics contribute to its notable antimicrobial and antioxidant activities. Furthermore, chitosan has garnered significant attention due to its non‐toxicity, antifungal and antimicrobial properties, biodegradability, biocompatibility, and antioxidant potential, which stem from its abundant amino and hydroxyl groups (Rabea et al. 2009). Recently, the commercial importance of chitosan has increased, largely because of the beneficial properties of its soluble derivatives.

The present review provides an overview of the recent trends in different applications along focusing on the importance and effects of water‐soluble chitosan derivatives, chitosan nano‐particles (CSNPs), chitosan conjugates, and chitosan Nano‐carriers on the plants, especially herbal plants, as well as on the main mechanisms of action. Additionally, in this paper, a large number of chitosan derivatives have been developed to produce improved materials with proposed or practical applications in agriculture and food systems, plant secondary metabolites and biochemistry, food technology, and industry.

## 2 The Main Characterization of Chitosan

### 2.1 Discovery and Physicochemical Properties of Chitosan

The discovery of chitosan dates back to 1811, when he isolated what he called “fungine” from fungal cell walls. About 30 years before the isolation of cellulose in 1823, a study on insects was conducted and found that the same structure was present in insects and plants. After that, chitosan was discovered in 1859 based on chitin deacetylation. The chemical structure of chitosan, however, was identified only in 1950 (Ferri and Tassoni 2011; Büyükyörük 2021).

### 2.2 Market Size

The global market for chitin and chitosan is projected to experience significant expansion in the upcoming years, driven by growing awareness of their benefits and sustainable nature. In 2022, the global market size of chitosan, recognized for its unique properties, was valued at USD 10 billion and is anticipated to reach USD 78.94 billion by 2032, growing at a compound annual growth rate (CAGR) of 23% due to its widespread industrial adoption (Iber et al. 2022; Hemmami et al. 2022). This growth is fueled by rising demand from various sectors, including agriculture, food, and medicine, contributing to the expansion of the global chitin market (Hahn et al. 2024).

The demand for chitin is also expected to rise in industries such as textiles, construction, biodegradable packaging, protective gear, and civil engineering over the next decade. Moreover, chitin and chitosan have garnered attention for their applications in nanotechnology, particularly in the production of polymer scaffolds (Martínez‐Cruz et al. 2021). Increased research and development efforts focusing on controlled cargo‐release mechanisms are anticipated to further accelerate global market growth in the near future. As the demand for natural and organic ingredients grows, chitin and chitosan have emerged as attractive options for food manufacturers seeking to improve the nutritional quality and ensure the safety of their products (Tavassoli et al. 2023). The antioxidant and antimicrobial properties of chitosan contribute to extending food shelf life, while its exceptional emulsifying capabilities enable it to replace synthetic surfactants in food technologies (Kaur et al. 2024). Furthermore, chitosan serves as a functional ingredient for addressing hypercholesterolemia, hypertension, and inflammation, and it supports nutrient encapsulation in functional food development. In 2021, revenue in the food and beverage sector reached $342.21 billion, underscoring the positive influence of such factors on the chitosan market's growth (Duan et al. 2022).

### 2.3 The Structure and Functional Properties of Chitin and Chitosan

The structure of chitosan is similar to cellulose, except for the acetamide group present at the C_2_ position (Wang et al. 2023). Chitosan can be extracted from chitin in several steps as follows: The process begins with extracting chitin from shrimp canning related to food industries; after that, the shrimp shells are crushed and ground into smaller particles, followed by the removal of calcium carbonate through demineralization and decalcification using diluted hydrochloric acid with continuous stirring. In the next stage, chitosan is obtained by subjecting the chitin to an alkali treatment (deproteinization), followed by deacetylation to convert it into chitosan (Egorov et al. 2022). Chemically, chitosan is a hydrophobic copolymer comprising *β* (1 → 4) linked D‐glucosamine repeating units and a small fraction (< 50 moL %) of *β* (1 → 4) linked N‐acetyl‐d‐glucosamine repeating units randomly distributed within the polymer (Duan et al. 2022). The functional groups allow researchers to modify the structure and properties of chitosan through various methods including grafting, crosslinking, composites, copolymerization, and molecular imprinting techniques, contained in chitosan, specifically amino (NH_2_), acetamido, and primary and secondary hydroxyl (OH) groups (Yadav et al. 2023; Argüelles‐Monal et al. 2018). The reactive amino group of chitosan has become particularly significant, as it facilitates diverse structural modifications. Physical modifications involve mixing and blending chitosan with other polymers, while chemical modifications include processes such as ionic interactions, crosslinking, grafting, and impregnation (Yadav et al. 2023). By changing the functional groups of chitosan, it is easy to achieve desired physicochemical properties. There are differences between chitin and chitosan; chitin is insoluble in most organic solvents and water regardless of the pH. Chitosan demonstrates a polycationic nature at pH levels below 6, due to the protonation of the amine groups and dissolves in aqueous acidic solutions (Wang et al. 2023). Generally, the solubility of chitosan is primarily influenced by the protonation of its amino groups, which occurs under acidic conditions. At acidic pH (typically below 6.0), the amino groups of chitosan become protonated, rendering the polymer positively charged and soluble in water. This solubility is essential for the preparation of chitosan‐based nanoparticles. However, as the pH increases above 6.0, the protonation decreases, leading to a loss of solubility and the formation of aggregates or precipitates. This pH‐dependent solubility is a fundamental characteristic of chitosan. The higher cationic charge can result in a strong electrostatic interaction, and it has also been demonstrated to be a biomaterial capable of transporting proteins and other active molecules. This interesting structural property of chitosan is due to the presence of a primary amine within the C‐2 position of the glucosamine residue, which allows it to efficiently react with aldehyde/ketone macromolecules (Piekarska et al. 2023; Jiménez‐Gómez and Cecilia 2020).

Chitosan has different applications compared to chitin, and these differences and abilities make it unique and attractive, such as having the ability of extensive hydrogen bonding and high charge density (Lingait et al. 2024). Another characteristic of chitosan is its higher molecular weight, which leads to reducing the hydrophilicity of gelatin films (Chen et al. 2023); chitosan also enhances the crosslinking of gelatin film through non‐covalent interactions with gelatin (Huang et al. 2023). Variation in chitosan applications can be due to things such as the origin of chitosan, length, pH, molecular weight, degree of deacetylation (DDA), degree of substitution (DS), and its functional groups. Although chitosan has versatile applications and properties, factors such as lower surface area, low thermal resistance, and insolubility in neutral and basic solvents limit its use. Therefore, physical or chemical modification of chitosan seems necessary (Harugade et al. 2023; Lingait et al. 2024).

### 2.4 Sources of Chitin and Chitosan

**FIGURE 1:**
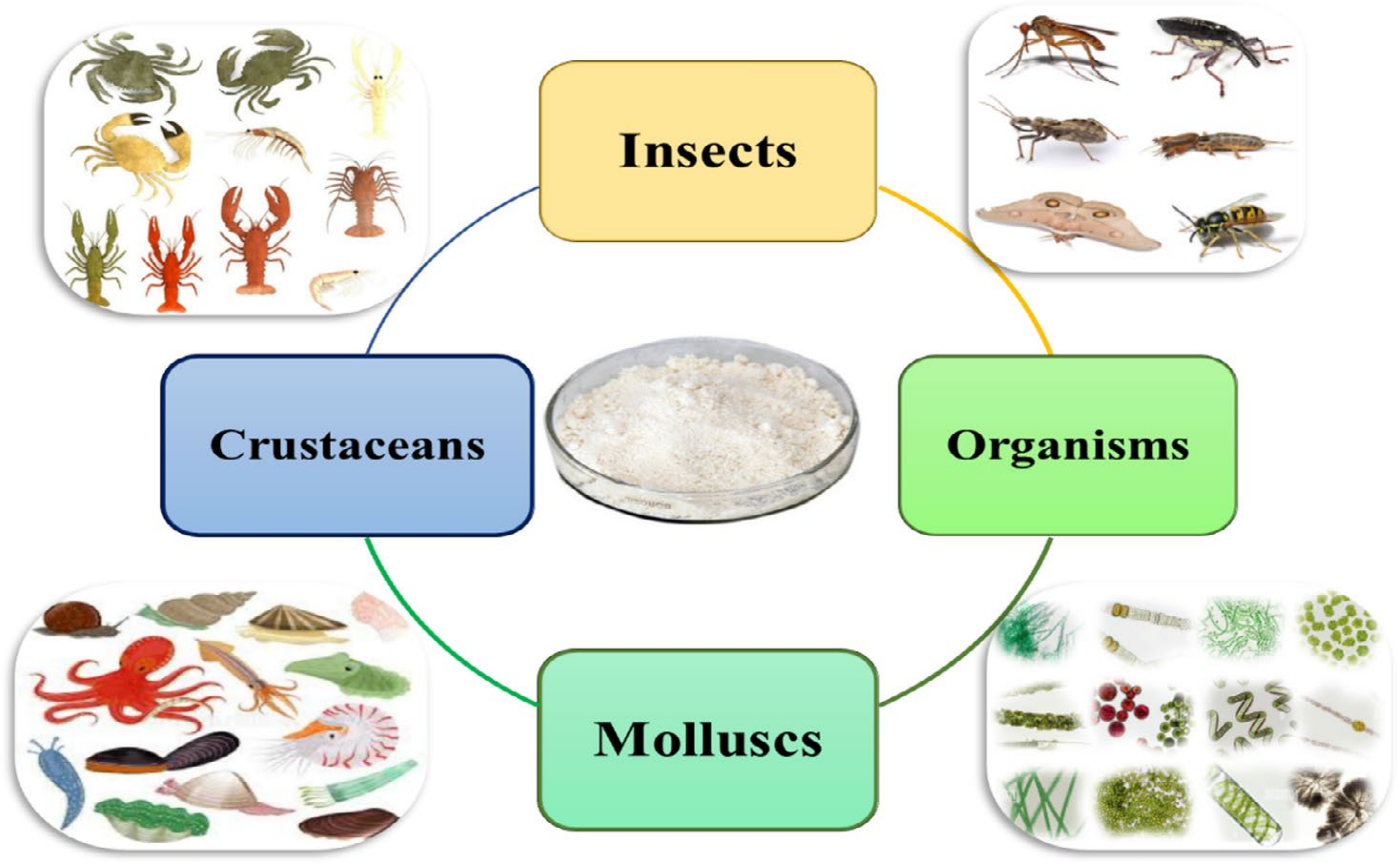
Various sources for chitin extraction and its subsequent conversion into chitosan.

Figure 1 illustrates various sources for chitin extraction and its subsequent conversion into chitosan. From the seafood traded annually, 6 to 8 million tons of crab, shrimp, and lobster shell waste are globally produced which contains 30%–40% calcium carbonate (CaCO_3_), 20%–40% protein, about 15% to 40% chitin, and a small amount of astaxanthin. Chitosan can be produced from chitin in crustacean shells such as oysters, shrimp, algae cell membranes, mollusk skeletons, and even plant cell walls. At present, the main source of chitosan is the shell of shrimp and crabs, and its content is about 30% of the volume of squid pens and fungi (Basawa et al. 2023).

A significant consideration is the sensitivity some individuals exhibit toward products derived from marine resources. Moreover, conventional chemical methods for extracting chitin from marine waste are costly and environmentally detrimental. Consequently, researchers are exploring alternative, sustainable sources, such as fungi and algae, for chitin extraction. Fungal cell walls are particularly noteworthy as a substantial chitin source, with studies indicating that optimizing cultivation conditions can significantly enhance chitin yields. In this context, extracting chitin from fungal mycelium, especially from *Aspergillus niger*, holds great importance. Advances in genetic engineering have further facilitated the manipulation of genes involved in chitin biosynthesis in *A. niger*, potentially increasing chitin deposition. Research also highlights that the cell walls of *P. pastoris* contain chitin, accounting for approximately 1%–5% (*w/w*) of the total cell wall composition. By leveraging genetic engineering techniques and overexpressing genes implicated in the chitin biosynthesis pathway, researchers can further augment chitin production. This approach even opens the possibility of using chitin as a by‐product of recombinant *P. pastoris* to develop high‐value proteins and other functional compounds (Zhang et al. 2023).

Traditionally, shrimp and crabs have been the most commonly referenced sources for chitosan extraction in scientific literature. However, other marine species, such as lobsters, crayfish, and oysters, have also been studied. The chitin content in crustaceans varies significantly across species. For instance, the shell waste of most crustaceans typically comprises 30%–50% calcium carbonate and 20%–30% chitin by weight. Notably, certain lobster species, including Nephrops and Homarus, exhibit remarkably high chitin content, ranging from 60% to 75%, the highest reported among chitin‐bearing organisms. Studies on extracting chitin and chitosan from crustacean by‐products, particularly those with a chitin content exceeding 20%, have demonstrated their viability as industrial feedstock. For example, by‐products from *Procambarus clarkia* (crayfish), including the whole body, thorax, and claws, contain approximately 20%–23% chitin by weight. The substantial chitin content, combined with the abundance and low cost of crayfish, makes it an economically attractive option for industrial‐scale chitin production. Furthermore, literature underscores the economic and ecological benefits of utilizing crustacean by‐products for chitosan production. An estimated 40%–50% of the total mass of crustaceans processed for human consumption is discarded as waste, much of which is improperly disposed of, contributing to coastal pollution. Consequently, crustacean by‐products, such as the cephalothorax of lobsters, represent a sustainable and promising source for large‐scale chitosan production, addressing both economic and environmental concerns (Ahmed et al. 2024).

### 2.5 Types of Chitosan

#### 2.5.1 Chitosan‐Plus

Chitosan‐Plus, an advanced derivative of chitosan, has garnered significant attention due to its superior properties and extensive applications across industries such as food and agriculture. This enhanced form is synthesized by modifying chitosan's molecular structure, often through chemical or enzymatic processes, to improve its solubility, bioactivity, and functional performance. Notably, Chitosan‐Plus demonstrates a higher degree of deacetylation, typically exceeding 90%, resulting in an elevated cationic charge density. This heightened charge facilitates stronger interactions with negatively charged microbial cell walls and surfaces, enhancing its antimicrobial efficacy. The improved properties of Chitosan‐Plus offer substantial advantages in both the food and agricultural sectors. In food preservation, it serves as a potent antimicrobial and antioxidant agent, extending the shelf life and ensuring the safety of food products. Within agriculture, its antifungal activity, plant growth‐promoting effects, and capacity to enhance soil health establish it as a valuable asset for sustainable farming practices (Kaviani Darani et al. 2025). The effectiveness of Chitosan‐Plus stems from its increased solubility, elevated cationic charge, and enhanced bioactivity, which collectively enable its beneficial interactions with biological systems and the environment (Ramya et al. 2012).

#### 2.5.2 Water‐Soluble Chitosan

Water‐soluble chitosan has been developed to enhance its solubility across a wide pH spectrum, achieved by incorporating hydrophilic groups such as carboxymethyl or hydroxypropyl functionalities. Unlike conventional chitosan, which dissolves only in acidic environments, water‐soluble chitosan remains stable and soluble in neutral and mildly alkaline conditions. This expanded solubility range makes it particularly suited for applications requiring consistent performance across varying pH levels. In the food industry, water‐soluble chitosan is valued for its preservative, health‐promoting, and functional attributes. Its water solubility enhances its interaction with other ingredients, improving its efficacy in preserving food and delivering health benefits. Similarly, in agriculture, water‐soluble chitosan serves as a bio‐pesticide, growth promoter, and a natural alternative to synthetic agrochemicals (Asghari Lalami et al. 2025). Its enhanced solubility contributes to improved bioactivity, making it more effective when applied to plants or soil systems (Jia et al. 2024). However, the inherent insolubility of traditional chitosan in water has limited its broader use, particularly in agriculture. To overcome this limitation, researchers have developed derivatives and oligomers through enzymatic and chemical modifications. These advanced biopolymers show significant potential for agricultural applications. Current research is also delving into the mechanisms of chitosan‐induced defense responses and signal perception in plants. Furthermore, the emergence of chitosan nanoparticles in nanotechnology offers exciting prospects, presenting innovative solutions for enhancing its functionality and expanding its applications (Sharp 2013).

#### 2.5.3 Low‐Molecular‐Weight (LMW) Chitosan

Low‐molecular‐weight (LMW) chitosan, characterized by its shortened polymer chains, has emerged as a highly promising material due to its superior solubility and bioactivity. Derived through the hydrolysis of standard chitosan into smaller molecular fragments, LMW chitosan exhibits enhanced functionality, surpassing that of high‐molecular‐weight chitosan. Typically ranging between 1000 and 100,000 Da, LMW chitosan contrasts sharply with conventional chitosan, which often exceeds molecular weights of 1,000,000 Da. This reduction in molecular weight significantly enhances its permeability and bioavailability, broadening its potential applications. The unique properties of LMW chitosan are intrinsically linked to its structural modifications. Increased solubility, resulting from its lower molecular weight, enables more effective interactions with biological systems. In the food sector, LMW chitosan demonstrates potent antimicrobial and antioxidant properties. Its mechanisms of action include disrupting microbial cell membranes and neutralizing free radicals, thereby enhancing food preservation and safety. In agriculture, LMW chitosan plays a dual role as a bio‐control agent and growth promoter. It activates plant defense pathways, stimulates the production of growth hormones, and improves soil microbial activity, collectively fostering plant health and resilience. This multifaceted functionality underscores its potential as a sustainable and effective alternative to synthetic chemicals in both food and agricultural industries (Tishchenko et al. 2011).

#### 2.5.4 Chitosan Derivatives

Chemical modifications of chitosan yield various derivatives, including N‐succinyl chitosan, carboxymethyl chitosan, and quaternary ammonium chitosan, each offering distinct advantages such as enhanced solubility, improved thermal stability, and specific functional properties. These modifications often result in derivatives with superior antioxidant activity or mucoadhesive capabilities, making them particularly valuable in food products. The functional efficacy of these chitosan derivatives in food and agriculture is primarily attributed to their increased solubility, pronounced cationic charge, and tailored chemical structures.

In agriculture, chitosan derivatives have gained prominence as effective tools for promoting plant growth, enhancing disease resistance, and serving as environmentally friendly alternatives to conventional synthetic pesticides and fertilizers. Their bioactivity and eco‐friendly nature align with the principles of sustainable farming, offering significant potential for reducing the environmental impact of agricultural practices (Alavi Samany et al. 2022). As ongoing research delves deeper into novel chitosan derivatives and their diverse applications, their role in fostering sustainable food production and agriculture is anticipated to expand substantially. These advancements underscore the versatility and importance of chitosan derivatives in addressing global food security and environmental sustainability challenges (Vinsova and Vavrikova 2011).

## 3 The Major Utilizations of Chitosan

### 3.1 Sustainable and Organic Agriculture Systems

In sustainable and organic agricultural systems, the incorporation of chitosan and its derivatives has proven to be effective in pest bio‐control and management, serving as a natural pesticide. Furthermore, it acts as a bacteriostatic and endophytic bio‐control agent for phyto‐pathogens and helps manage weed growth. Chitosan's application also enhances photosynthesis, respiration, and plant metabolism, fostering plant growth, promoting nutrient uptake, and improving the nutritional value of crops (Alavi Samany et al. 2022). These beneficial effects contribute to the long‐term sustainability of biomass production and yield, while simultaneously enhancing the quality of output in both horticultural and agronomic crops (Wang et al. 2024; Emami Bistgani et al. 2017).

Chitosan emerges as a highly versatile and environmentally friendly bio‐stimulant, holding considerable promise for transforming agricultural practices. Its capacity to bolster disease resistance, improve growth, and alleviate the effects of abiotic stress positions it as a valuable asset in promoting sustainable crop production (Alavi Samany et al. 2022). Additionally, chitosan's role in extending the shelf life of produce and mitigating post‐harvest losses underscores its pivotal contribution to food security and the economic viability of agriculture. By addressing contemporary challenges and leveraging advanced technologies, the full potential of chitosan can be fully realized to meet the growing demands of modern, sustainable agriculture by enhancing soil fertility, promoting plant growth, and serving as a growth regulator, bio‐pesticide, and disease resistance agent (Shahrajabian et al. 2021; Giglou et al. 2022).

#### 3.1.1 Photosynthetic Activity

Chitosan plays an integral role in enhancing the photosynthetic processes of plants by elevating chlorophyll levels, optimizing antioxidant enzyme activity, and safeguarding the photosynthetic apparatus from damage. Research indicates that the application of chitosan results in the enhancement of shoot height, node development, membrane stability, and an increase in the content of chlorophyll, carotenoids, proline, and sugars, all of which collectively improve the photosynthetic efficiency of plants (Sun et al. 2023). Additionally, chitosan is involved in photochemical processes, contributing to the exploration of mechanisms that depend on sunlight for essential biochemical activities. It plays a key role in the chlorophyll fluorophore, a crucial indicator of the initiation of photosynthetic activity. The application of chitosan has proven effective in enhancing shoot growth, promoting node formation, reinforcing membrane integrity, and increasing the content of vital biochemical compounds such as chlorophyll, carotenoids, proline, and sugars (Alavi Samany et al. 2022).

#### 3.1.2 Activation of Plant Defense Responses

Chitosan acts as an elicitor, initiating the expression of defense‐related genes in plants (Riseh et al. 2024). This activation triggers the production of phytoalexins, pathogenesis‐related proteins, and various antimicrobial substances, thereby strengthening the plant's immune defenses. As a result, plants exhibit enhanced resilience to a broad spectrum of pathogens, including fungi, bacteria, and viruses (Magnabosco et al. 2023). Chitosan demonstrates extensive antimicrobial activity against pathogens such as *Botrytis cinerea*, *Fusarium oxysporum*, and *Pseudomonas syringae* . By reducing the incidence of disease, it helps minimize crop losses and reduces dependence on chemical pesticides, supporting the principles of sustainable agriculture (Magnabosco et al. 2023). Furthermore, chitosan fortifies the structural integrity of plant cell walls by promoting the biosynthesis of cellulose and lignin, creating a robust physical barrier that hinders pathogen infiltration and proliferation, thereby providing an additional layer of protection against microbial threats (Ramírez‐Rodríguez et al. 2024).

#### 3.1.3 Hormonal Modulation

Chitosan influences hormonal pathways that regulate essential plant growth hormones, including auxins, gibberellins, and cytokinins. This hormonal interaction supports harmonious plant growth and enhances stress adaptability. Furthermore, chitosan stimulates the activity of antioxidant enzymes, mitigating oxidative damage and promoting overall plant vitality (Ghosh et al. 2020). As a plant growth regulator, chitosan improves the efficiency of reactive oxygen species elimination, strengthens plant membranes, and contributes to higher crop yields. It fosters enhanced branching, restricts excessive shoot development, and promotes fruit growth in a variety of plants (Shahrajabian et al. 2021). The porous nature of chitosan facilitates the efficient movement of water and nutrients, thereby boosting plant nutrient uptake. Thus, chitosan, in synergy with plant growth regulators, plays a pivotal role in augmenting plant productivity.

#### 3.1.4 Improved Growth and Yield

Chitosan stimulates the development of roots and shoots, thereby improving nutrient absorption and photosynthetic efficiency (Riseh et al. 2024). These improvements contribute to increased biomass accumulation, enhanced flowering, and higher yields, ultimately resulting in greater agricultural productivity (Ungureanu et al. 2023). Previous studies have shown that foliar application of chitosan enhances growth parameters and biomass yields in various agronomic and horticultural crops, including peppermint, tomato, wheat, hyssop, chamomile, basil, and lemon balm (Goudarzian et al. 2020; Giglou et al. 2022).

#### 3.1.5 Abiotic Stress Mitigation

Chitosan plays a critical role in enhancing plant resilience to environmental stressors such as drought, salinity, and extreme temperature fluctuations. It regulates stomatal behavior, reduces transpiration rates, and improves water‐use efficiency, thereby aiding plants in maintaining homeostasis under adverse conditions. Furthermore, its ability to preserve chlorophyll content during periods of stress contributes to sustained growth and promotes recovery (Duan et al. 2019). Environmental stressors including drought, salinity, extreme temperatures, and pathogen infestations significantly impact global agricultural productivity. Addressing these challenges necessitates innovative approaches to improve plant resilience. Among the numerous strategies explored, chitosan—a natural biopolymer derived from the deacetylation of chitin—has shown considerable promise. Its effectiveness in enhancing plant tolerance to environmental stress is particularly evident when applied as a foliar spray in agricultural settings or as an elicitor in tissue and cell culture systems (Vosoughi et al. 2018; Giglou et al. 2022). Chitosan's biocompatibility, biodegradability, and non‐toxic nature make it an ideal, eco‐friendly solution for modern agricultural practices (Rezaei‐Adl et al. 2025). In this context, chitosan can activate antioxidant defense mechanisms, mitigating toxicity caused by abiotic stresses and making it an attractive option for agricultural applications. The physiological effects of chitosan on plants vary depending on factors such as species, chitosan concentration, and the developmental stage of the plant. Chitosan can be applied in several forms, including seed coatings (for crops like soybean, cotton, wheat, and rice), soil enrichment, foliar sprays (for peanut, soybean, rice, maize, cotton, and others), and as a supplement in hydroponic systems (for rice, wheat, and peanuts). One of the key challenges to the widespread use of chitosan in agriculture lies in the standardization of commercial chitosan products and issues related to its solubility. The bioactivity of chitosan is influenced by a range of factors, including its degree of acetylation, molecular weight, concentration, pH levels, viscosity, and the specific microorganisms being targeted (Desai et al. 2023; El Nahrawy et al. 2020; Emami Bistgani et al. 2017). Additionally, studies have shown that the antimicrobial efficacy of chitosan is closely linked to its molecular weight, with lower molecular weights generally leading to enhanced antibacterial activity (Fan et al. 2023). However, establishing a definitive relationship between molecular weight and bioactivity remains a subject of ongoing research. The foliar application of chitosan has gained significant attention due to its direct impact on enhancing plant stress tolerance (Vosoughi et al. 2018; Emami Bistgani et al. 2017; Giglou et al. 2022; Alavi Samany et al. 2022; Riseh et al. 2024; Rezaei‐Adl et al. 2025; Asghari Lalami et al. 2025).

Chitosan enhances the activity of key antioxidant enzymes, including superoxide dismutase (SOD), catalase (CAT), and peroxidase (POD). These enzymes play a crucial role in mitigating oxidative stress induced by the excessive production of reactive oxygen species (ROS) during abiotic stresses such as drought and salinity (Rezaei‐Adl et al. 2025). By scavenging ROS, chitosan reduces cellular damage and bolsters plant resilience (Magnabosco et al. 2023). Water deficit stress, a major factor contributing to reduced agricultural and horticultural productivity, induces oxidative stress by disrupting the balance between ROS generation and the plant's antioxidant defense mechanisms. Antioxidants are molecules that neutralize reactive oxygen species, thereby preventing damage to plant cells (Mosaedi et al. 2024). Chitosan's ability to scavenge ROS and improve plant performance under environmental stresses has garnered substantial interest, resulting in its diverse applications in agricultural systems (Rezaei‐Adl et al. 2025). Notably, chitosan has shown potential as an antiperspirant agent, enhancing water deficit tolerance by bolstering oxidative stress defense without compromising crop yield (Almeida et al. 2020; Shinde et al. 2024).

Chitosan, acting as an elicitor, stimulates plant defense signaling pathways involving key molecules such as salicylic acid, jasmonic acid, and ethylene (Ghasemi Pirbalouti et al. 2017; Ahmed et al. 2024; Shinde et al. 2024; Riseh et al. 2024). This activation induces the expression of stress‐responsive genes, resulting in the synthesis of protective proteins and secondary metabolites that enhance the plant's resilience to both biotic and abiotic stresses. Furthermore, chitosan influences stomatal activity, minimizing water loss through transpiration (Riseh et al. 2024).

Chitosan's role extends beyond foliar applications to tissue and cell culture systems, where it functions as an elicitor, enhancing stress tolerance and stimulating secondary metabolite production. These controlled environments maximize the benefits of chitosan, offering key advantages (Shahrajabian et al. 2021).

Chitosan induces the synthesis of secondary metabolites, including phenolics, flavonoids, and alkaloids, which function as antioxidants. These compounds protect plant cells from oxidative damage while enhancing their defense mechanisms (Ghasemi Pirbalouti et al. 2017; Ahmed et al. 2024; Shinde et al. 2024; Riseh et al. 2024; Asghari Lalami et al. 2025).

In tissue culture, chitosan plays a pivotal role in facilitating callus induction and organogenesis by modulating hormonal levels and cellular differentiation. This function is crucial for regenerating plants with increased stress resistance and advancing biotechnological applications.

Chitosan treatment stimulates the activation of stress‐responsive genes, leading to the synthesis of protective proteins such as heat‐shock proteins (HSPs) and pathogenesis‐related (PR) proteins. These proteins are integral to strengthening plants against various environmental stresses (Ali et al. 2021; Mehmood et al. 2020).

Chitosan significantly promotes somatic embryogenesis, a crucial process for large‐scale plant propagation and genetic enhancement. This capability is indispensable for developing cultivars that are resilient to stress, thereby addressing key challenges in agricultural sustainability. The efficacy of chitosan is markedly increased when combined with other compounds, such as amino acids—essential organic molecules that are integral to various physiological and biochemical processes in both agriculture and food systems. The synergistic interaction between chitosan and these compounds enhances productivity while also contributing to the preservation and improvement of food quality, highlighting the potential of integrated strategies to advance sustainable practices in these fields (Pongprayoon et al. 2022).

#### 3.1.6 Controlled‐Release Fertilizers

Chitosan is increasingly utilized in controlled‐release fertilizers (CRF) to mitigate fertilizer pollution in agriculture. By incorporating chitosan into controlled‐release microspheres, which are enriched with nitrogen, nutrients are gradually released into the soil, thereby enhancing fertilizer efficiency and minimizing nutrient loss. The performance of CRF is influenced by several factors, including the composition of the coating materials, the specific type of CRF employed, environmental conditions, and agricultural practices. These variables collectively determine the rate and effectiveness of nutrient release. A variety of preparation methods, such as emulsification, crosslinking, and nanoparticle synthesis, are employed to produce CRF. The controlled‐release mechanism relies on the gradual diffusion of nutrients through the coating material, with factors such as the size and morphology of the nanocomposites, as well as the physical form of the CRF, playing a significant role in governing the nutrient release rate (Mujtaba et al. 2020).

#### 3.1.7 Pesticide Removal and Environmental Protection

The substantial increase in agricultural productivity is largely attributed to the extensive use of pesticides globally. However, despite the widespread application of pesticides, crop yields continue to be reduced by about 40% due to the consistent presence of pests, insects, weeds, and plant pathogens (Pathak et al. 2023). Chitosan derivatives have also found significant utility in the removal of pesticides, such as acephate, orthostats, and methyl parathion, from contaminated water sources. These derivatives exhibit impressive adsorption capacities and follow pseudo‐first‐order kinetics. Moreover, fungicide resistance poses a serious challenge to agriculture, with chitin deacetylase emerging as a novel target for the development of agricultural fungicides aimed at combating pathogens like *Podosphaera xanthii*, the causative agent of cucurbit powdery mildew (Martínez‐Cruz et al. 2021).

Chitosan has gained prominence as an effective solution for mitigating abiotic stress in plants, especially in the face of challenges such as climate change, urbanization, and the depletion of natural resources within the agricultural sector (Campos et al. 2023; Nguyen et al. 2023). One of the key applications of chitosan lies in wastewater treatment and water purification. This eco‐friendly biopolymer proves highly efficient in removing a wide range of contaminants, including heavy metals, radioactive substances, dyes, pesticides, fertilizers, antibiotics, biological contaminants, and oil and grease wastes from polluted water sources (Crini et al. 2019a; Pal et al. 2021).

#### 3.1.8 Synergistic Effects in Agriculture Systems

The combination of chitosan with other bioactive compounds, such as amino acids, has demonstrated remarkable synergistic effects, particularly in agricultural applications. Amino acids, as essential organic molecules, are integral to a broad range of physiological and biochemical processes in plants, including protein synthesis, enzyme regulation, and the modulation of stress responses. When integrated with chitosan, these compounds function synergistically to enhance plant health, stimulate growth, and improve resilience to environmental stressors. The observed synergism can be primarily attributed to the complementary mechanisms of action of both chitosan and amino acids. Chitosan, recognized for its elicitor activity, induces plant defense responses by activating genes associated with pathogen resistance and stress adaptation. In parallel, amino acids act as precursors for key metabolic pathways, providing the essential substrates required for the synthesis of proteins, secondary metabolites, and compounds involved in stress responses. Beyond promoting growth and enhancing resistance, the chitosan–amino acid combination also improves nutrient uptake efficiency (Chitranshi et al. 2020; Ungureanu et al. 2023).

Amino acids play a crucial role in chelating essential micronutrients, enhancing their bioavailability and mobility within plant systems. Chitosan, with its film‐forming properties, promotes the controlled‐release and absorption of these nutrients, thereby optimizing their delivery. This interaction not only improves the nutritional profile of plants but also reduces the dependency on excessive chemical fertilizers, supporting sustainable agricultural practices. Moreover, this synergistic approach effectively alleviates abiotic stresses, such as drought, salinity, and extreme temperatures (Vosoughi et al. 2018; Emami Bistgani et al. 2017; Giglou et al. 2022; Riseh et al. 2024).

The benefits of chitosan and amino acids extend beyond cultivation into post‐harvest management. Their combined antimicrobial and antioxidative properties provide an effective strategy for maintaining the quality and extending the shelf life of harvested produce. By reducing microbial spoilage and oxidative damage, this combination preserves the freshness, marketability, and nutritional value of perishable crops (Mehmood et al. 2020).

### 3.2 Improving of Secondary Metabolites in Medicinal Herbs

Plants have been utilized for their medicinal properties since antiquity. The pharmacological attributes of plants are attributed to their phytochemical constituents, particularly a diverse group of organic compounds known as plant secondary metabolites. These metabolites, derived from primary metabolites, serve as significant sources of bioactive compounds with various physiological functions (Lavanya et al. 2020; Najafabadi et al. 2023; Larijanian et al. 2024). Plants of economic importance produce a wide array of organic compounds, including alkaloids, polyphenols, essential and fixed oils, resins, gums, tannins, saponins, natural rubber, waxes, dyes, and various specialty products (Hamedi et al. 2022; Memarzadeh et al. 2020, 2024). Secondary metabolites play a crucial role in facilitating the interaction between plants and their environment, contributing to plant survival and fitness. These metabolites are as essential to plants as primary metabolites; some derivatives, such as brassinosteroids, gibberellins, and strigolactones, are critical for plant growth and function (Waratadar et al. 2021; Maghsoudi et al. 2023). Moreover, plant secondary metabolites are responsible for the characteristic taste, odor, and color of plants, providing defense against pathogens, herbivores, and environmental stressors (Costa et al. 2012; Momeni et al. 2020).

Extensive research has been conducted on the production of secondary metabolites, particularly in terms of biosynthesis pathways and genetic modifications aimed at meeting the growing demands of high‐value agricultural ecosystems. Plant secondary metabolites are employed in various industries, including agrochemicals, flavoring, food additives, nutrition, pharmaceuticals, cosmetics, fragrances, bio‐pesticides, natural dyes, and biotechnology (Shinde et al. 2024). These bioactive compounds are utilized therapeutically (e.g., codeine, atropine, and cardenolides) (Rashid and Rashid 2022), as insecticides (e.g., laurine, chlorobutanol), hallucinogens (e.g., morphine, scopolamine, tetrahydrocannabinol) (Batool et al. 2023), antioxidants (e.g., carsonic acid, rosemary oil), and as flavors and fragrances (e.g., capsaicin, vanillin, rose, and lavender oils). Based on the biosynthetic pathway, the plant secondary metabolites are classified into three major groups: terpenes, phenolic compounds, and nitrogen‐containing compounds (alkaloids, glucosinolates, and cyanogenic glycosides) (Shinde et al. 2024).

One of the prominent approaches in the production of secondary metabolites involves the use of elicitors within cell, organ, and plant tissue culture technologies. Elicitors, categorized into chemical and biological types, are critical compounds that stimulate physiological changes and promote the accumulation of phytoalexins. These substances play a pivotal role in enhancing the production of secondary metabolites (Shinde et al. 2024).

Given the economic significance and growing demand for secondary metabolites, researchers are increasingly focused on strategies to enhance both the quantity and quality of these compounds. Modern biotechnological approaches, such as cell culture, tissue, and organ cultures, alongside the use of biological elicitors like chitosan, offer promising solutions for boosting secondary metabolite production. Elicitors of active ingredients play a crucial role in triggering defense responses and elevating the yield and quality of secondary metabolites (Alavi Samany et al. 2022). They also influence gene expression in plants, either by overcoming enzyme inhibition or by altering enzymatic pathways, thus modifying the production levels of these compounds (Momeni et al. 2023). Moreover, the active components of biological elicitors significantly contribute to stimulating defense mechanisms and augmenting the synthesis of secondary metabolites (Shinde et al. 2024).

Chitosan, a highly effective biological elicitor, has been widely recognized for its ability to enhance the production of secondary metabolites in various medicinal plants. While the exact mechanism through which chitosan exerts its effects on plants remains unclear, the cultivation of plant cells to extract valuable bioactive compounds remains a key area of interest among researchers. In this section, we examine the role of chitosan in stimulating secondary metabolite production in selected medicinal plants and examine its influence on their overall growth performance (Vosoughi et al. 2018; Alavi Samany et al. 2022; Rezaei‐Adl et al. 2025).

In a study, *Silybum marianum* cell suspension cultures were treated with seven distinct concentrations of chitosan. The results demonstrated that chitosan enhanced both biomass production and silymarin accumulation. Silymarin, the key compound in *S. marianum* , is a mixture of isomeric flavonolignan analogs, including silycristin, silybins, isosilybins, silydianin, and taxifolin. This bioactive compound is known for neutralizing oxidative damage caused by free radicals, thereby offering protective benefits to human liver tissue (Shah et al. 2021). In another study by Mohammadi et al. (2021), chitosan was applied in three stages to two oregano species ( *Origanum majorana* and *Origanum vulgare* ) under water deficit stress. The findings revealed that water deficit stress reduced plant dry weight but increased essential oil and total phenolic content. However, foliar application of chitosan under these conditions resulted in increased dry weight of shoots and enhanced phenolic compound levels. Saleh et al. (2023) reported that foliar application of chitosan nanoparticles improved mycorrhizal colonization in wheat, promoting plant growth, physiological processes, and root colonization. This effect was linked to a 32% increase in auxins and a 21% increase in strigolactones in the roots, which are key phytohormones involved in plant development. This study also highlighted the positive impact of chitosan on the efficiency of arbuscular mycorrhizal fungi. Additionally, a study investigating the effects of chitosan as an elicitor at various concentrations on the physiological and biochemical properties of stevia under salinity stress found that salinity stress reduced chlorophyll (Chl *a*, Chl *b*, and total), carotenoids, and total protein content. However, foliar spraying of chitosan under saline conditions improved the levels of the main active compounds, stevioside and rebaudioside A, while also reducing malondialdehyde content and enhancing the activity of antioxidant enzymes, such as catalase and peroxidase (Gerami et al. 2020).

Fooladi Vanda et al. (2019) examined the effects of varying chitosan concentrations on the antioxidative capacity, enzyme activities involved in the defense response of lemon balm ( *Melissa officinalis* L.), and the biosynthesis of rosmarinic acid. Their findings revealed that chitosan treatment significantly enhanced the activity of key enzymes such as phenylalanine ammonia‐lyase (PAL), catalase (CAT), guaiacol peroxidase (GPX), and lipoxygenase (LOX). Furthermore, the expression of PAL1, TAT, and RAS genes was upregulated, resulting in increased accumulation of rosmarinic acid and phenolic compounds in treated shoots. The study concluded that chitosan‐induced production of rosmarinic acid is facilitated by the activation of defense‐related enzymes, the upregulation of TAT and RAS gene expression, and the stimulation of jasmonic acid biosynthesis (Fooladi Vanda et al. 2019). In another investigation by Forouzandeh et al. (2019), the impact of chitosan as an elicitor to replace chemical fertilizers on fennel ( *Foeniculum vulgare* L.) growth, enzyme activity, and yield was explored. The results demonstrated that chitosan treatment improved photosynthetic pigment levels, increased the concentration of soluble osmolytes, and provided protection against damage from electrolytic leakage, ultimately leading to enhanced fruit yield. Additionally, chitosan application resulted in higher yield components and promoted the biosynthesis of secondary metabolites in the plant (Forouzandeh et al. 2019).

A comprehensive review of previous studies (Ghasemi Pirbalouti et al. 2017; Vosoughi et al. 2018; Bistgani et al. 2017; Momeni et al. 2020; Alavi Samany et al. 2022; Rezaei‐Adl et al. 2025; Asghari Lalami et al. 2025; Kaviani Darani et al. 2025) indicated that the foliar application of chitosan has proven effective in enhancing the production of secondary metabolites, including essential oils, in several medicinal and aromatic plants cultivated in Iran. These herbs, including *Ocimum ciliatum*, *O. basilicum* , *Thymus daenensis*, *Thymbra spicata*, *Salvia officinalis* , *Mentha × piperita*, *Hyssopus angustifolius*, *Achillea mille folium*, *Mentha spicata* , and *Capsicum annum*, exhibited improved essential oil yields when treated with chitosan. The alteration in the composition of biologically active compounds, such as essential oils, is believed to result from changes in phytohormonal balance or modifications in the structural tissues, including glandular trichomes responsible for secretion.

### 3.3 Chitosan Coatings: A Sustainable Approach to Preserving Herbal Products

The application of chitosan coatings has emerged to prolong the shelf life and preserve the quality of herbal products. This natural biopolymer not only acts as a protective shield against environmental stressors but also demonstrates inherent antibacterial properties, making it an effective solution for safeguarding these high‐value commodities. Recent advances show that combining chitosan with plant‐derived starches and bioactive essential oils can create multifunctional protective films capable of regulating gas exchange, minimizing moisture loss, and stabilizing key phytochemicals in herbal materials. These hybrid coatings utilize both the intrinsic antimicrobial activity of chitosan and the added antioxidant potential of natural extracts, offering a sustainable alternative to synthetic preservatives for sensitive botanical products (Lingait et al. 2025). The following sections delve into the mechanisms and advantages of using chitosan coatings in the preservation of herbal products. However, despite the numerous benefits, there are challenges that must be addressed to facilitate the widespread adoption of chitosan coatings (Chitranshi et al. 2020). One such challenge is the integration of essential oils into chitosan coatings, which, while enhancing antimicrobial and antioxidant properties, may also alter the sensory characteristics of the final product, including its taste and aroma. Consequently, meticulous formulation is required to strike a balance between antimicrobial effectiveness and consumer preference. The incorporation of nanotechnology into chitosan coatings offers promising potential for more precise control over the release of active compounds. Furthermore, standardizing chitosan formulations for specific uses and ensuring compliance with food and pharmaceutical regulations are critical steps for achieving commercial success and gaining broad market acceptance (Sahariah and Másson 2017). The mechanisms by which chitosan coatings contribute to extending shelf life and maintaining the quality of herbal products are outlined below.

#### 3.3.1 Barrier Properties

Chitosan coatings are widely acknowledged for their exceptional barrier properties, especially in regulating the permeability to gases, water vapor, and solutes. These characteristics are vital in retarding the oxidation process, a primary contributor to the deterioration of herbal products, while also curbing moisture loss that can lead to dehydration. The efficacy of chitosan coatings is significantly influenced by several environmental factors, such as temperature, relative humidity, and light exposure. For example, under high humidity conditions, the hydrophilic nature of chitosan may compromise its barrier effectiveness against moisture. Conversely, in environments with lower humidity, chitosan coatings typically maintain their structural integrity, efficiently preventing excessive dehydration of herbal products (Thambiliyagodage et al. 2023). Temperature fluctuations further affect both the mechanical strength and the barrier properties of chitosan films, as higher temperatures may soften the coating and diminish its protective capabilities. When compared to ambient conditions, herbal products coated with chitosan display superior preservation qualities, as the coating provides a protective shield against UV radiation. Changes in relative humidity and temperature are often responsible for oxidative degradation and microbial contamination, but the chitosan coating serves as a protective buffer, maintaining a stable microenvironment that safeguards the volatile compounds and active ingredients in herbs, which are particularly vulnerable to degradation under adverse conditions. Furthermore, the preservation efficacy of chitosan coatings can be enhanced by modifying the chitosan formulation. Alterations such as adjusting the degree of deacetylation, incorporating plasticizers, or combining chitosan with other natural substances can improve the mechanical properties and barrier performance of the films, thereby making them more suitable for maintaining the quality of herbs across a range of storage environments (Ghosh et al. 2020).

#### 3.3.2 Antimicrobial Activity

The antimicrobial properties of chitosan are attributed to its cationic nature, which facilitates interactions with the negatively charged membranes of microbial cells. This interaction disrupts the integrity of the cell membrane, resulting in the leakage of intracellular contents and ultimately causing cell death (Ghosh et al. 2020). Extensive research has substantiated the effectiveness of chitosan coatings in extending the shelf life of various herbal and plant‐based products. For example, chitosan‐coated guava slices were able to retain their freshness and quality for up to 17 days, whereas uncoated samples deteriorated after just 3 days (Hanani et al. 2023). In another study, the encapsulation of essential oils such as carvacrol, eugenol, and thymol in chitosan not only stabilized their bioactive compounds but also facilitated their controlled‐release during storage. Additionally, the synergistic application of chitosan and essential oils ensures an extended shelf life without compromising the sensory qualities of herbal products, including their aroma and flavor profiles (Thambiliyagodage et al. 2023).

#### 3.3.3 Antioxidant Potential

The antioxidant properties of chitosan are principally ascribed to its capacity to scavenge free radicals and reactive oxygen species, both of which contribute significantly to oxidative stress. Free radicals, such as hydroxyl and superoxide radicals, are highly reactive molecules that can inflict damage upon cellular components, including lipids, proteins, and DNA. In the case of herbal products, oxidation can result in the degradation of volatile compounds, essential oils, and active ingredients, thereby diminishing the therapeutic and sensory attributes of the herbs. Chitosan's ability to neutralize these free radicals effectively mitigates oxidative damage, thus preserving the functional properties of the herbs over time (Thambiliyagodage et al. 2023).

The antioxidant efficacy of chitosan is contingent upon several factors, including its degree of deacetylation, molecular weight, and the presence of additives. Chitosan with a higher degree of deacetylation generally exhibits enhanced antioxidant activity, as this modification increases the number of free amino groups available for interaction with free radicals. Furthermore, the molecular weight of chitosan influences its solubility and its ability to form networks, which, in turn, affects the accessibility of its antioxidant sites. Additionally, chitosan has been shown to produce synergistic effects when combined with other natural antioxidants, such as polyphenols or essential oils, further augmenting its ability to protect against oxidative degradation. Therefore, incorporating chitosan into herbal product formulations offers a dual function—serving both as a physical barrier and as an antioxidant agent (Duan et al. 2019).

#### 3.3.4 Benefits in Post‐Harvest Storage

Chitosan's antimicrobial and antioxidant properties render it an exceptional choice for edible coatings on fruits and vegetables. The coating creates a semi‐permeable barrier that minimizes moisture loss, regulates gas exchange, and curtails microbial activity, thereby preserving the freshness and nutritional integrity of produce throughout storage. By mitigating spoilage and decay, chitosan substantially reduces post‐harvest losses, thereby enhancing the marketability of perishable goods. This not only contributes to food security but also provides significant economic advantages to farmers and distributors (Sun et al. 2023).

### 3.4 The Application of Chitosan Food Systems

**FIGURE 2:**
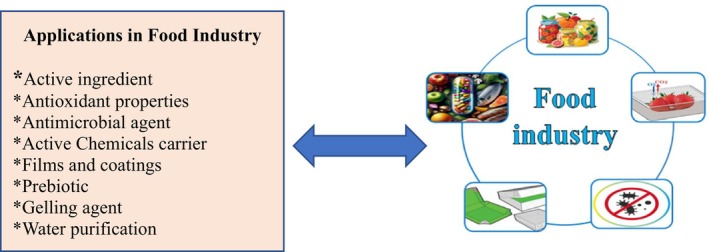
A summary of the general applications of chitin and chitosan in the food industry.

Chitosan is a highly versatile molecule with a broad array of potential applications in food technology and industry, including processing, food storage, preservation, food stabilization, animal feed additives, anti‐cholesterol agents (fat traps), as well as in veterinary and medical fields (Crini et al. 2019b; Akhtar et al. 2023). A comprehensive overview of the general uses of chitin and chitosan within the food industry is illustrated in Figure 2. Notably, chitosan and its derivatives are biodegradable, biocompatible, non‐toxic, and renewable. These attributes, combined with its partial solubility in acetic acid and hydrochloric acid, enhance its ability to form films (Ding and Guo 2022).

The primary applications of chitosan in the food and fragrance industries include: (1) as a filler and food additive in products such as cheese, sauces, soups, canned goods, nuts, biscuits, sweets, bread, and chips, where it plays a crucial role in prolonging the shelf life of food products while inhibiting the growth of bacteria and fungi; (2) as an antioxidant, antibacterial, antifungal, anti‐allergic, and anti‐inflammatory agent in the pharmaceutical industry; (3) as a filler in plastic production; (4) as a filler and waterproofing agent in paper and paper product manufacturing; (5) as an antibacterial and odor‐neutralizing agent in the production of clothing and synthetic fibers; and (6) for its antioxidant, anti‐aging, and antibacterial properties in the formulation of cosmetic products such as anti‐aging creams, shampoos, soaps, antiseptic creams, and skin protectors (Table 1) (Ghasemi Pirbalouti et al. 2017; Fernando et al. 2024; Fatima et al. 2024; El‐Araby et al. 2024).

**TABLE 1:** Addresses the applications of chitin and chitosan in the food and agricultural industries.

Topic	Applications	Material	References
Agriculture	Defensive inducing mechanism in plants	Chitin	Riseh et al. (2024)
Agriculture and Water Engineering	For decontaminated plutonium‐containing wastewater and water‐containing methylmercury acetate	Chitin	Sun et al. (2023)
Agriculture	Induce favorable changes in the metabolism of plants and fruits	Chitin and Chitosan	Kertmen et al. (2023)
Food industry	Creating texture in meat and cheese products	Copolymer of chitosan with methyl methacrylate, polyurea‐urethane, poly (amide ester), acrylamide anhydride	Ibrahim et al. (2023)
Food industry	Prebiotic sausage formulation with β‐glucan such as Noticeable effect on physician and sensory properties	Chitin and Chitosan	Shahbaz et al. (2023)
Food industry	Dairy products with β‐glucan such as Calorie‐reduced and cholesterol‐lowering	Chitin and Chitosan and β‐glucan	Riseh et al. (2024)
Food industry	Yogurts with β‐glucan such as Faster proteolysis, lower release of large peptides and a higher proportion of free amino acids	Chitosan and CMC and β‐glucan	Shahbaz et al. (2023)
Food industry	Extruded ready‐to‐eat snacks such as Manipulate the glycemic response	Chitin and Chitosan and agar gums and β‐glucan	Thambiliyagodage et al. (2023)
Food industry	Beverage containing β‐glucan such as Control food intake and reduce 24 h energy intake	Chitosan and Inulin and β‐glucan	Zhang et al. (2023)
Agriculture	Resistance against Botrytis cinerea in *Solanum lycopersicum*	Chitosan	Kumari and Kishor (2020)
Agriculture	Yield, grain protein, iron, and zinc contents in *Triticun aestivum*	Nano‐Chitosan	Riseh et al. (2024)
Agriculture	Resistance against *Fusarium pseudograminearum* and *Fusarium graminearum* in *Triticun aestivum*	Nano‐chitin	Sun et al. (2023)
Agriculture	Growth promotion and improved disease resistance and increase of plant growth and resistance against *Pyricola grisea*	Chitosan‐based nanoparticles complexes with metals (Zn, Cu, …)	Kertmen et al. (2023)
Food industry (packaging)	An increase in the total plasticizer content resulted in a considerable decrease of tensile strength (up to 50% of the original values when 30% plasticizer was added) An increase in the total plasticizer content resulted in a considerable decrease of elasticity modulus	Chitosan–gelatin plasticized with water or polyols	Riseh et al. (2024)
Food industry (packaging)	Evaluated an against *S. enteritidis* in suspensions. When applied directly to cell suspensions, 1% chitosan reduced the pathogen > 4 log10 CFU/mL (or 99.99%).	Chitosan films combined with various ingredients	Srinivasa and Tharanathan (2007)

#### 3.4.1 Packaging System

The packaging system plays a crucial role in extending the shelf life of fresh and processed food products beyond their natural expiration while preserving their quality, nutritional value, and sensory attributes. Chitosan has emerged as a widely utilized biomaterial in food packaging (Table 2) (Al‐Maqtari et al. 2022; Fernando et al. 2024; Fatima et al. 2024).

**TABLE 2:** Refers to the applications of chitin and chitosan in cosmetic, pharmaceutical, and health products.

Materials	Topic	Forms	Application	References
Chitosan	Styling Gel	Gels	Thickened aqueous system Non‐abrasive No foaming agents Compatible with relevant antimicrobials Specific devices are needed for applications	Thambiliyagodage et al. (2023)
Chitosan	Chewing gum/lozenges	Solid preparation	Larger contact time Stimulated salivary secretion Useful for patients with low tooth brushing compliance	Kertmen et al. (2023)
Chitosan	Sustained‐release formulations/devices	Solid preparation	Long‐term effect The efficacy is independent of patient compliance	Ibrahim et al. (2023)
Chitin	Dentifrices	Gel form toothpastes	Complex formulation Possible interaction among components Tooth brushing with a dentifrice is a well‐adopted habit	Piekarska et al. (2023)
Chitin	Body Deodorant Spray	Gas	The ratio of mass to volume (Rho). Relatively small doses to achieve efficacy Good compliance Easy usage	El‐Araby et al. (2024)
Chitosan	Hair care products requisites Improved combability	Liquid and Gel form	Low stickiness Lack of powdering or flaking Preferably being clear Preferably transparent Preferably glossy Good film formation Good holding power High level of style retention Prolonged curl retention Easily removed upon washing the hair	Rachtanapun et al. (2023)
N, N‐Di carboxymethyl chitosan	Oral healthcare	Liquid and Gel form	A particular delivery mechanism reduce bacterial adsorption on S‐HA in vitro Dose dependent effect Better activity on S. mutants reduce bacterial adsorption on S‐HA in vitro Dose dependent effect Better activity on S. mutants	Kankariya and Chatterjee (2023)
Chitosan	Drug delivery	solid preparation	encapsulation of sensitive drugs: to increase and to modulate drug release rate Controlled drug delivery carriers	Kumar et al. (2023)
Chitin	Ophthalmology Oral drug delivery	Sponge Nanoparticle	Biological adhesive: water‐resisting adhesive In situ gelation Transfection Permeation enhancement Healing: wound healing, self‐healing Pump inhibitory properties Products for radiopharmaceutical domains Nutraceutical ingredients	Rossi et al. (2024)
Chitosan and alginate	Drug delivery Textile finishes Scaffold for nerve tissue regeneration	Capsule, microcapsule	Chemical and enzymatic deproteinization; demineralization; characterization; deacetylation; biological activities; biomedical applications	Younes and Rinaudo (2015)
Chitosan and CMC	Orthopedics Contact lenses Bacteriostatic agent Hemostatic agent	Powder	Dietary antioxidants, which inactivate reactive oxygen species and provide protection from oxidative damage	Jasim (2021)
Chitosan	Skin delivery system	Capsule, microcapsule	By loading the active ingredients ensuring their penetration through the skin, and targeting and releasing them at the right dose and site, thus improving their bioavailability and efficacy with minimal side effects	Aranaz et al. (2014)
Chitin	Wound dressings for the treatment of deep skin wounds	Nanofibrils films, and sponges	This material prevents the penetration of pathogens from the external environment onto the surface of the wound chitosan under the action of a biologically active medium and the extracellular matrix resorbs without adversely affecting the surrounding tissues and organs	Shahbaz (2020)

The adoption of bio‐based polymeric materials in the packaging industry presents significant potential, primarily driven by the environmental and human health concerns linked to petroleum‐based packaging materials. Technological innovations have facilitated the development of chitosan‐based films with diverse functionalities, positioning them as a promising alternative for packaging applications (Priyadarshi and Rhim 2020). Chitosan‐based packaging, celebrated for its biocompatibility, biodegradability, antibacterial properties, and low cytotoxicity, has been found in various packaging domains, including antibacterial films, barrier films, and sensor films. However, substantial efforts remain necessary to refine chitosan‐based films to meet practical requirements and effectively compete with conventional petroleum‐based packaging materials. Chitosan‐based packaging for food products is categorized into two distinct types: active and intelligent packaging, each of which will be discussed separately (Rostamabadi et al. 2024).

Chitosan plays an essential role in active packaging, contributing significantly to the extended shelf life of food products due to its unique properties. Active packaging involves the intentional inclusion of functional components within packaging materials, which can either release or absorb substances to enhance shelf life and maintain food quality (Kaya et al. 2022). While chitosan‐based scaffolds offer numerous benefits, they are limited by certain mechanical shortcomings, including low tensile strength, poor fracture stiffness, rapid degradation, and restricted osteoconductivity. Numerous studies have demonstrated that the mechanical properties of chitosan films can be affected by several factors, such as the degree of deacetylation of chitosan, molecular weight, the method used in film production, chitosan concentration, storage time, and the specific environmental conditions under which testing is performed, including the solvent type used for dissolving the chitosan. Due to its hydrophilic nature, chitosan films exhibit relatively low water barrier properties (Bajić et al. 2020). The water vapor permeability of these films is governed by factors like molecular weight, degree of deacetylation, and chitosan concentration (Mannucci et al. 2024).

Furthermore, external factors such as the measurement technique, environmental conditions, and storage time can significantly impact the results. To improve the barrier properties, essential oils and lipid compounds have been incorporated into the chitosan matrix. However, it is important to note that the addition of essential oils does not lead to a consistent enhancement of these properties across all conditions. Additionally, chitosan's relatively low thermal stability poses a major challenge to its use as a biodegradable material on an industrial‐scale. To address this, efforts have been made to improve the thermal stability of chitosan by incorporating various plasticizers, such as water, ethylene glycol, glycerol, and sorbitol (Srinivasa and Tharanathan 2007). The inclusion of these plasticizers enhances the flexibility, stretchability, and workability of chitosan films, mitigating some of the material's inherent limitations. Furthermore, to reduce chitosan's heightened sensitivity to humidity, it is often combined with other bio‐based materials, such as gelatin, collagen, casein, whey protein, zein, and essential oils. Pure chitosan films, however, exhibit relatively low radical scavenging activity, primarily due to the absence of hydrogen atoms that can effectively act as antioxidants. Moreover, the weak free radical scavenging capacity of the free amino groups at the C‐2 position of chitosan chains further contributes to the suboptimal antioxidant activity of chitosan films (Bi et al. 2019). To enhance the antioxidant properties of natural active compounds, such as essential oils and polyphenols, have been incorporated into the chitosan matrix through physical mixing, resulting in the production of composite films.

For comparative analysis, Table 3 presents the antioxidant property values of different enriched chitosan‐based films. Additionally, the functional properties of the films include antimicrobial activity (Aworunse et al. 2024). The antibacterial efficacy of chitosan is governed by several factors, including the degree of deacetylation, molecular weight, film‐forming conditions, pH, temperature, and other variables. Extensive studies have shown that chitosan effectively inhibits the growth of a wide array of fungi and bacteria, with greater potency against Gram‐positive bacteria compared to Gram‐negative strains. As a result, the integration of chitosan into active packaging has shown considerable promise in prolonging the shelf life of diverse food products (Bajić et al. 2020; Rostamabadi et al. 2024).

**TABLE 3:** Antioxidant properties of various chitosan‐based films.

Film composition	Antioxidant agent	Test method	Antioxidant activity (%)	Effectiveness range	Potential applications	References
Chitosan–Grape Seed Extract	Grape Seed Extract	ABTS Assay	78.2 ± 1.8	Moderate	Food packaging, pharmaceuticals	Ghoshal and Singh (2024)
Chitosan–Curcumin	Curcumin	Ferric Reducing Antioxidant Power (FRAP)	92.1 ± 3.1	Very High	Anti‐inflammatory drugs, skin protection	Jiang et al. (2024)
Chitosan–tannic acid	Tannic acid	DPPH Radical Scavenging	70.5 ± 1.5	Moderate	Ladyfinger packaging	Kurabetta et al. (2023)
Chitosan–Chitooligosaccharide‐Caffeic Acid Conjugate	Chitooligosaccharide‐Caffeic Acid Conjugate	ABTS Assay	82.3 ± 2.4	High	Active packaging	Yuan et al. (2023)
Chitosan–Summer Savory Essential Oil	Summer Savory Essential Oil	FRAP	75.8 ± 2.0	Moderate	Active packaging	Atlar et al. (2024)
Chitosan–Red Poppy Anthocyanins	Red Poppy Anthocyanins	DPPH Radical Scavenging	80.1 ± 2.5	High	Colorimetric sensor for shrimp freshness	Tavassoli et al. (2023)

In contrast, intelligent packaging refers to materials designed to monitor the condition of packaged food products or their surrounding environment. These packages relay information about the status of the food without directly interacting with it (Sarfraz et al. 2024). The primary goal of intelligent packaging is to perform advanced functions, such as detecting, recording, tracking, and communicating data (Kaya et al. 2022; Khan et al. 2024; Sarfraz et al. 2024). These functions aim to extend the food's shelf life, enhance its quality, and provide alerts regarding potential issues. Examples of intelligent packaging include time–temperature indicators, gas leakage sensors, and humidity or freshness indicators. A specific category of intelligent packaging, based on chitosan, can be classified into two main types (Khan et al. 2024): first, films that exhibit visual color changes through colorimetric reactions, and second, sophisticated biosensors, such as novel antifungal peptides identified through functional screening of marine metagenomic samples. The purified recombinant peptide demonstrated inhibitory effects against *Candida albicans* and *Aspergillus niger*. The first group of chitosan‐based intelligent packaging includes indicators for time, temperature, freshness, or pH levels. This category has been extensively discussed in the literature on food packaging materials. Intelligent pH indicator films, for example, monitor the freshness of food products by changing color in response to pH variations (Ramakrishnan et al. 2024). This allows consumers to quickly assess the quality of the food they are about to consume. Research on chitosan‐based pH indicator films has been particularly focused on fishery products. For instance, studies have shown that incorporating alizarin into chitosan films causes a dramatic color shift from yellow to purple within a pH range of 4–10. Furthermore, the addition of butterfly pea extract to chitosan films leads to a color transition from purple‐blue to dark green during fish preservation (Wu et al. 2024).

The increasing reliance on petroleum‐based polymers in food packaging has raised serious environmental concerns and highlighted the urgent need for their replacement with biodegradable alternatives. Among these, chitosan has attracted significant attention due to its biocompatibility, non‐toxicity, film‐forming ability, and inherent antibacterial and antifungal activities. Although pure chitosan films exhibit certain limitations in terms of mechanical strength and barrier properties, studies have demonstrated that their tensile strength can be enhanced and water vapor permeability reduced by incorporating polyphenols and nanoparticles. Likewise, the addition of essential oils and plant extracts has been shown to markedly improve the antioxidant and antimicrobial capacity of chitosan‐based films. For instance, an increase in DPPH radical scavenging activity to over 90% and a notable reduction in water vapor permeability in the presence of gallic acid and TiO_2_ have been reported. Such modifications enable the design of active packaging systems capable of controlling microbial growth and oxidative processes. On the other hand, the development of intelligent chitosan‐based films incorporating natural pigments such as anthocyanins and curcumin has provided efficient tools for monitoring food freshness and detecting spoilage, where color changes triggered by pH variations or amine production can visually indicate product quality. Moreover, the integration of chitosan with metallic or semiconductor nanoparticles has enabled the fabrication of time–temperature indicators and volatile compound sensors. The growing number of scientific publications over the past decade reflects the increasing interest in these technologies and the shift toward their industrial application, despite persistent challenges such as standardization of testing protocols, safety evaluation of active compound migration, and production scalability. Overall, chitosan and its reinforcing components represent a promising platform for the development of active and intelligent packaging systems that can simultaneously address food safety requirements and environmental sustainability.

#### 3.4.2 Chitosan Nanoparticle (CS‐NPs)

Chitosan nanoparticles, as biocompatible and biodegradable drug carriers, have broad applications in targeted drug delivery, tissue engineering, vaccination, and antimicrobial therapy. Various methods exist for the synthesis of these nanoparticles.

Ionotropic gelation is one of the most common and straightforward methods for CS‐NP synthesis. In this method, a chitosan solution is mixed with a negatively charged crosslinking agent such as tripolyphosphate (TPP) to form nanoparticles. The process can be represented as follows:

$$\begin{array}{c} \mathrm{CS}-\mathrm{NH}\;2+\mathrm{TPP}\;3-\to \mathrm{CS}-\mathrm{TPP}\;\text{nanoparticles}\;\mathrm{CS}\\ \;-\mathrm{NH}\;2+\mathrm{TPP}\;3-\to \mathrm{CS}-\mathrm{TPP}\;\text{nanoparticles} \end{array}$$

In this reaction, free amino groups of chitosan interact with TPP ions, forming a three‐dimensional network that results in nanoparticle formation. Parameters such as pH, chitosan and TPP concentrations, temperature, and reaction time significantly influence particle size and distribution. Studies have shown that this method produces nanoparticles with uniform sizes and high drug loading capacity (Des Bouillons‐Gamboa et al. 2024).

In nanoprecipitation, a chitosan solution in an organic solvent is slowly added to a non‐solvent solution, leading to nanoparticle formation:

$$\mathrm{CS}\;\left(\text{solvent}\right)\to \mathrm{CS}\;\left(\text{precipitate}\right)\;\mathrm{CS}\;\left(\text{solvent}\right)\to \mathrm{CS}\;\left(\text{precipitate}\right).$$

The addition to the non‐solvent decreases chitosan solubility, triggering nanoparticle precipitation. This method is suitable for generating uniform nanoparticles with high drug encapsulation efficiency (Jayan et al. 2025).

In the emulsion‐extrusion method, a chitosan solution in an organic solvent is added to an aqueous phase containing an emulsifier to form an emulsion. The emulsion is then passed through a membrane with defined pore size to yield nanoparticles:

$$\begin{array}{c} \mathrm{CS}\;\left(\text{solvent}\right)+\text{Emulsifier}\to \text{Emulsion}\to \mathrm{CS}-\mathrm{NPs}\;\mathrm{CS}\;\left(\text{solvent}\right)\\ \;+\text{Emulsifier}\to \text{Emulsion}\to \mathrm{CS}-\mathrm{NPs} \end{array}$$

This process enables the production of nanoparticles with uniform size and high drug loading capacity and provides enhanced colloidal stability.

In the microfluidic method, chitosan solution and crosslinker are combined at high flow rates through microchannels, resulting in nanoparticle formation:

$$\begin{array}{c} \mathrm{CS}\;\left(\text{fluid}\right)+\text{Crosslinker}\;\left(\text{fluid}\right)\to \mathrm{CS}-\mathrm{NPs}\;\mathrm{CS}\;\left(\text{fluid}\right)\\ \;+\text{Crosslinker}\;\left(\text{fluid}\right)\to \mathrm{CS}-\mathrm{NPs}. \end{array}$$

Rapid mixing within microchannels produces nanoparticles with uniform and controllable physicochemical properties, suitable for applications requiring precise particle characteristics.

A comprehensive review (Tian et al. 2023) has mentioned the design and synthesis of chitosan‐based nanoparticles (CS‐NPs) for cancer drug delivery. It emphasizes chemical modifications—including PEGylation, folate conjugation, and hydrophobic crosslinking—and the fabrication of chitosan‐nanocomposite systems (e.g., with ZnO, Ag, or graphene oxide) to enhance circulation stability, drug encapsulation efficiency (EE), and controlled‐release. Results of case studies demonstrate that targeted modifications, such as folate or peptide ligands, can increase cellular internalization in receptor‐overexpressing cells by several‐fold, with some reports achieving EE for hydrophobic drugs exceeding 80%–90%. The review also highlights stimuli‐responsive strategies (pH, enzymatic, thermal) for localized release and combinatorial therapies (chemotherapy plus immunotherapy or phototherapy). Nevertheless, it underscores persistent challenges, including inconsistent EE measurement protocols, colloidal stability in serum, and limited quantitative long‐term toxicity data in animal models.

In a research‐focused review (Kesharwani et al. 2024), the authors address folate‐functionalized chitosan nanoparticles as targeted anticancer carriers. Folate conjugation serves as a targeting ligand for folate receptors, which are overexpressed in many cancer cells, enhancing selectivity and cellular uptake. Both in vitro and in vivo studies demonstrate that this modification significantly improves tumor‐to‐normal tissue selectivity while reducing systemic toxicity. Animal data show enhanced therapeutic efficacy, with tumor volume reductions of 40%–70% in specific models, alongside improved pharmacokinetic profiles. The authors, however, emphasize the need for comprehensive safety evaluations and organ‐specific bio‐distribution studies in larger preclinical models.

In another review (Stefanache et al. 2025), current methods for chitosan nanoparticle (CS‐NP) preparation—including ionotropic gelation, emulsion‐crosslinking, nanoprecipitation, and self‐forming techniques—and categorizes loading efficiency, release kinetics, and stability for various drugs have been compared. Practical insights indicate that ionotropic gelation is the simplest and least cytotoxic method, with EE ranging from 40%–90% depending on the drug and controllable particle sizes of 50–400 nm. The review also discusses specialized delivery applications, including ocular, nasal, and oral routes, and provides evidence of improved bioavailability for poorly water‐soluble drugs via CS‐NPs. Simultaneously, it highlights shortcomings such as limited long‐term stability data and the lack of standardized preclinical reporting.

Manna et al. (2023) reported that positively charged chitosan derivatives effectively interact with cellular membranes via electrostatic interactions, offering advantages in gene and nucleic acid delivery (e.g., for lentiviral systems). PEGylation improves circulation stability and reduces clearance by the reticuloendothelial system. The article specifically addresses challenges in achieving homogeneous drug loading and production consistency, providing technical recommendations for clinical translation, such as quality control measures and standardized toxicity testing.

#### 3.4.3 Functional Foods and Nutraceuticals

Natural products, particularly plant‐derived nutraceuticals, are increasingly recognized for their potential as functional ingredients in food, offering promising therapeutic applications for conditions such as cancer, diabetes, bacterial infections, and other diseases (Picos‐Corrales et al. 2023). In this context, chitosan‐based colloidal carriers have been developed to safeguard these bioactive compounds, yielding micro‐ and nano‐particles that are both biocompatible and biodegradable (Wang et al. 2024). Potato peel waste represents a rich source of bioactive polyphenols with antioxidant, anti‐inflammatory, and anticancer properties, offering potential benefits in the prevention of various chronic diseases. These valuable compounds can be efficiently recovered using biopolymers such as chitosan, enabling their incorporation into nutraceutical formulations. Although the adsorption capacity of chitosan is inherently limited, surface modification or grafting strategies can enhance functional sites, thereby improving polyphenol uptake and overall efficacy (Lingait et al. 2024). The interactions between chitosan, an amino polysaccharide, and nutraceutical compounds are primarily driven by hydrogen bonding and electrostatic forces. Notably, studies have shown that the electrostatic interaction between curcumin and chitosan nanoparticles is closely correlated with the number of intermolecular hydrogen bonds formed (Hu and Luo 2021; Ni et al. 2022). Several investigations have focused on developing nutraceutical formulations for oral administration utilizing chitosan and its modified derivatives. For example, a study by Anter et al. (2019) demonstrated that apocynin, an anti‐inflammatory compound found in *Apocynum cannabinum* roots, could be encapsulated with an efficiency of 45%, offering controlled drug release under in vitro gastrointestinal conditions.

The authors also noted that this carrier system provided excellent stability and enhanced efficacy during prolonged oral administration, particularly for treating gastric ulcers. Similarly, Kamal et al. (2021) developed nanogels from chitosan grafted with *ρ*‐coumaric acid and loaded with *Syzygium aromaticum* essential oil. These nanogels exhibited improved antioxidant activity and significantly enhanced the antibacterial properties of the native oil. In another study, Garcia‐Carrasco et al. (2022) compared chitosan to copolymers made from chitosan and polyethylene glycol methyl methacrylate, incorporated with phenolic compounds extracted from Mexican oregano ( *Lippia graveolens* ). The copolymer system provided superior protection for the active agents under simulated gastric conditions. Furthermore, Moon et al. (2021) reported that encapsulating quercetin in platforms composed of chitosan and soybean polysaccharides led to the formation of nanoparticles specifically targeting disease‐related cells. This formulation demonstrated enhanced stability and improved bioactivity, including antioxidant, anticancer, and anti‐inflammatory effects. The encapsulated quercetin also exhibited better bioavailability and solubility in aqueous solutions compared to its free form. Recent studies have also highlighted the potential of nano‐chitosan encapsulated onion extract as a protective agent with cancer‐preventive and anti‐tumor properties, suggesting that it could serve as a synergistic alternative to traditional cancer chemotherapy. Additionally, chitosan derived from fungi has garnered increasing attention in the market due to its appeal to vegan consumers.

#### 3.4.4 Synergistic Application of Chitosan and Amino Acids in the Food Industry

The synergistic effects between chitosan and amino acids stem from their complementary biochemical and structural characteristics. Chitosan's polycationic nature facilitates its interaction with negatively charged molecules, such as amino acids, forming stable complexes that exhibit enhanced functional properties. These complexes contribute to improved solubility, bioavailability, and overall efficacy within food systems. In particular, the molecular interactions between chitosan and amino acids significantly enhance the structural integrity and functional performance of films and coatings. To optimize these benefits, future research should aim to fine‐tune the ratios and application methods for specific crops and food products. Additionally, the use of advanced technologies such as nanotechnology and encapsulation could further bolster the stability and efficacy of chitosan‐amino acid formulations (Xie et al. 2024).

Chitosan's capacity to form edible films and coatings is crucial for extending the shelf life of perishable food items. Its inherent antimicrobial properties effectively curtail the growth of spoilage organisms, while its barrier function minimizes moisture loss and prevents oxidative degradation. The addition of amino acids, such as lysine and glutamine, to chitosan‐based coatings further amplifies their antioxidant potential, thereby preserving food quality. This synergistic combination has shown exceptional efficacy in prolonging the freshness of fruits, vegetables, and seafood. Moreover, the dual action of chitosan's antimicrobial effects and the antioxidant properties of amino acids provide robust protection against both microbial spoilage and oxidative damage. This is particularly vital for lipid‐rich foods, which are especially susceptible to oxidative rancidity. Amino acids like cysteine, known for their potent antioxidant capabilities, enhance the preservative functions of chitosan, thereby improving the shelf life and safety of food products (Chen et al. 2023).

Chitosan‐based coatings can be enriched with amino acids to combat nutritional deficiencies and enhance the overall nutritional profile of food products. For instance, incorporating essential amino acids such as lysine and methionine not only improves the nutritional value but also boosts the functional properties of the chitosan matrix. These fortified formulations are pivotal in the creation of functional foods aimed at promoting human health and well‐being (Wang et al. 2011).

The synergistic combination of chitosan and amino acids has proven effective in the development of biodegradable food packaging materials. Chitosan provides the necessary structural integrity for film formation, while amino acids improve the films' flexibility, transparency, and mechanical strength. Moreover, these films can be engineered to release amino acids gradually, functioning as active packaging solutions that ensure the preservation of food safety and quality over prolonged periods (Rkhaila et al. 2021).

## 4 Challenges and Future Perspectives

Despite its many benefits, the efficacy of chitosan is influenced by factors such as plant species, environmental conditions, and application techniques. Key determinants of its performance include molecular weight, degree of deacetylation, and concentration. Future research should focus on optimizing chitosan formulations tailored to the needs of specific crops and stress conditions. Innovations in nanotechnology and encapsulation methods hold the potential to enhance chitosan's delivery and bioavailability. Moreover, exploring alternative, cost‐effective sources of chitin, such as insect biomass, may provide solutions to the economic challenges associated with producing high‐quality chitosan. At the molecular level, a deeper understanding of chitosan's interaction with plant signaling pathways is crucial. Identifying specific receptors and their downstream targets could pave the way for the development of genetically modified crops that exhibit enhanced responsiveness to chitosan treatments (Jia et al. 2024). Moreover, chitosan nanoparticles may have genotoxic effects. Results of previous research (De Lima et al. 2010; Taner et al. 2014) indicated chitosan particles were toxic only at larger sizes and higher concentrations. Overall, smaller chitosan particles can be used safely at higher concentrations than large particles. In a review evaluation (Frigaard et al. 2022) on chitosan free and chitosan nanoparticles found that the majority of chitosan nanoparticles demonstrated low cytotoxicity regardless of particle composition, derivatives, cytotoxicity assay, cell lines and animals used in both in vitro and in vivo studies. According to the results of documented reports on chitosan toxicity, the cytotoxicity evaluation should still be performed for all new chitosan‐containing nanoparticles in the presence of food packaging films on gene expression and on biological indicators of oxidative stress to obtain additional information concerning the safety of these polymeric nanoparticles and their impacts on humans and on the environment.

## 5 Conclusions

Biopolymers, particularly chitin and its derivative chitosan, are significant macromolecules sourced from various living organisms, including crustaceans, fungi, and plants. Chitin, the second most abundant biopolymer after cellulose, is primarily extracted from the exoskeletons of marine invertebrates, which constitute a substantial portion of seafood waste. This waste, often discarded, presents a valuable opportunity for chitin recovery, as it contains significant amounts of this biopolymer. Chitosan, derived from chitin, possesses unique physicochemical properties due to its functional groups, making it a versatile material with applications across multiple industries, including food, pharmaceuticals, and agriculture. Recent trends highlight the increasing commercial value of chitosan. Chitosan's role in agriculture systems is particularly noteworthy, as it enhances plant growth, improves disease resistance, and mitigates abiotic stresses. Its ability to stimulate plant defense mechanisms and antioxidant systems positions chitosan as a crucial bio‐stimulant for sustainable agricultural practices. Furthermore, developing various chitosan derivatives, such as water‐soluble and low‐molecular‐weight chitosan, expands its applicability and effectiveness in different environmental conditions. The ongoing research into optimizing chitosan extraction from sustainable sources, including fungi and algae, alongside advancements in its modification, underscores its potential in addressing modern agricultural challenges. As the demand for eco‐friendly agricultural inputs grows, chitosan's multi‐functionality and biodegradability will likely play a pivotal role in shaping future agricultural systems, thereby contributing to enhanced food security and environmental sustainability.

## Author Contributions

**Maryam Moslehishad:** writing – review and editing, supervision, investigation, resources. **Saeedeh Karimlar:** writing – Original Draft, Research. **Abdollah Ghasemi Pirbalouti:** conceptualization, Writing – Review and Editing. **Zahra Teymuori:** writing – Original Draft, Research. **Maryam Moslehishad:** conceptualization, Supervision, Writing – Review and Editing. **Zohreh Hamidi‐Esfahani:** review and Editing.

## Conflicts of Interest

The authors declare no conflicts of interest.

## Data Availability

The data that support the findings of this study are available from the corresponding author upon reasonable request.
